# Visually targeted reaching in horse-head grasshoppers

**DOI:** 10.1098/rspb.2012.0918

**Published:** 2012-07-04

**Authors:** Jeremy E. Niven, Swidbert R. Ott, Stephen M. Rogers

**Affiliations:** 1 Department of Biology and Environmental Sciences, School of Life Sciences, University of Sussex, Falmer, Brighton BN1 9QG, UK; 2 Department of Zoology, University of Cambridge, Downing Street, Cambridge CB2 3EJ, UK

**Keywords:** sensorimotor integration, reaching, limb control, vision, distance perception, motor control

## Abstract

Visually targeted reaching to a specific object is a demanding neuronal task requiring the translation of the location of the object from a two-dimensionsal set of retinotopic coordinates to a motor pattern that guides a limb to that point in three-dimensional space. This sensorimotor transformation has been intensively studied in mammals, but was not previously thought to occur in animals with smaller nervous systems such as insects. We studied horse-head grasshoppers (Orthoptera: Proscopididae) crossing gaps and found that visual inputs are sufficient for them to target their forelimbs to a foothold on the opposite side of the gap. High-speed video analysis showed that these reaches were targeted accurately and directly to footholds at different locations within the visual field through changes in forelimb trajectory and body position, and did not involve stereotyped searching movements. The proscopids estimated distant locations using peering to generate motion parallax, a monocular distance cue, but appeared to use binocular visual cues to estimate the distance of nearby footholds. Following occlusion of regions of binocular overlap, the proscopids resorted to peering to target reaches even to nearby locations. Monocular cues were sufficient for accurate targeting of the ipsilateral but not the contralateral forelimb. Thus, proscopids are capable not only of the sensorimotor transformations necessary for visually targeted reaching with their forelimbs but also of flexibly using different visual cues to target reaches.

## 1. Introduction

Many insect species show impressive capabilities in behavioural tasks often assumed to be challenging even for many large-brained vertebrates despite having much smaller and simpler nervous systems [1]. For example, bumblebees are capable of predicting the timing of future events based on past events [2], while honeybees can learn sameness–difference rules [3], and paper wasps (*Polistes fuscatus*) can use facial patterns to distinguish between conspecifics [4]. That insects can perform these behaviours challenges the intuition that sophisticated behavioural capabilities necessarily require large numbers of neurons. Moreover, because insect nervous systems have comparatively few neurons, they provide opportunities for research into the neural circuits and processing algorithms that generate these behaviours. Last, but not least, characterizing the diverse repertoire of behavioural capabilities among insects against the backdrop of their specific ecologies provides insights into the selective pressures that lead to their evolution [5]. Although many studies have focussed on the behavioural capabilities of the insects in the context of sensory systems, and learning and memory processes, few have focussed on their capabilities in the context of motor control.

A demanding sensori-motor control problem that has been studied intensively in mammals, including humans and cats, is the visual targeting of limb movements. Mammals use visually targeted limb movements to reach for nearby objects and to find footholds while walking [6,7]. Targeting a limb requires that the target object's location is encoded by the visual system in retinotopic co-ordinates, which must then be transformed into a co-ordinated activation of motor neurones that moves the limb to the target (for a review see [8]). Without vision, animals have to rely upon rhythmic searching movements to systematically sample space around the limb and use the ensuing mechanosensory feedback to find the target; not only does it take longer to locate objects in this way, it may also draw unwelcome attention to an animal trying to remain camouflaged. Although targeted reaching has been suggested to have evolved from the visual placement of forelimbs during walking [6], these are distinct behaviours. Visual limb placement while walking involves the modification of an ongoing motor pattern and is restricted to particular phases of the stepping cycle to prevent instability. Moreover, visual placement in walking animals involves small adjustments to a limb's trajectory within a restricted space. By contrast, targeted reaches are more typically made by stationary animals and involve movement of a limb to a location anywhere in the visual field that is within range. That insects can use vision to aid limb placement while walking was recently shown in locusts [9]. Vision influences limb placement in many other insect behaviours [10,11], but it is unknown whether insects are capable of direct, targeted reaching to objects located anywhere in visual space.

**Figure 1.**
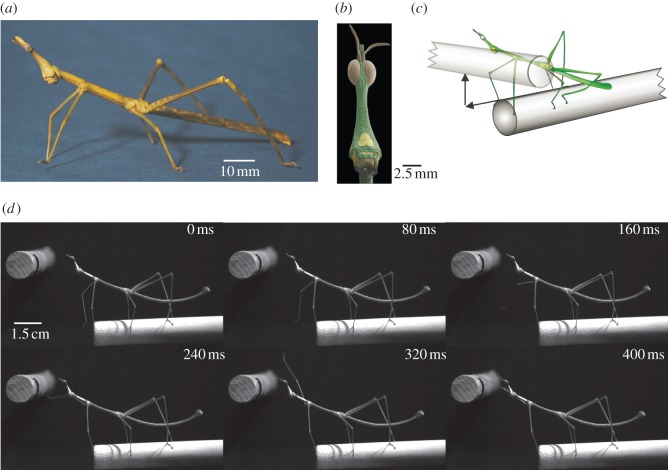
Visually targeted forelimb movements in proscopids. (*a*) *Pseudoproscopia scabra* resembles a stick insects in its external morphology. (*b*) A scanning electron micrograph of the head of *Prosarthria teretrirostris.* (*c*) The arrangement of the rods for producing gaps of variable vertical and horizontal sizes. (*d*) A high-speed video sequence of *Ps. scabra* reaching across a 2 cm vertical gap.

Here, we show that horse-head grasshoppers (Acridoidea: Proscopididae) use vision to accurately reach directly to locations in their environment without making sweeping and searching movements. Horse-head grasshoppers are related to other grasshoppers (Acrididae) but superficially resemble stick insects (Phasmatodea); they are wingless with long thin limbs and a narrow tubular thorax and abdomen (figure 1*a*) [12]. Proscopids differ from stick insects in having prominent near-holoptic compound eyes which, as their family name suggests (proscopia ‘looking out for’), are positioned atop their elongated heads (figure 1*b*). Stick insects detect gaps and locate footholds by sweeping their long antennae rhythmically through the space in front of them [13]. The antennae of proscopids, however, are very short and thus cannot be used in this way (figure 1*b*). Their only options for detecting gaps and locating footholds would therefore seem to be undirected sampling movements made with their forelimbs or visual information. We show that stationary proscopids rely exclusively upon visual inputs when reaching for targets within a large region of space in front of them. Our evidence suggests that they rely on inputs from frontal eye regions of binocular overlap to target their limbs to nearby locations, but when locations are more distant or binocular overlap regions are occluded, the proscopids switch to peering to obtain distance information from motion parallax. Thus, our findings demonstrate that an insect nervous system is capable of flexibly transforming visually encoded target positions within a large space into accurate limb motor trajectories that bring the tarsus directly into contact with the target.

## 2. Material and methods

### (a) Animals

Adult male horse-head grasshoppers (*Pseudoproscopia scabra* Klug [14] and *Prosarthria teretrirostris* Brunner Von Wattenwyl [15]) were selected at random from colonies maintained in the Department. The two species are extremely similar in morphology, differing principally in size (male *Pr. teretrirostris* are about 70 mm in total body length, *Ps. scabra* are about 90 mm long).

### (b) Measuring regions of binocular overlap

The frontal binocular overlap between the two compound eyes of six male proscopids was measured using a Zeiss goniometer. The heads of proscopids were fixed using bees' wax in a hole made in the centre of a glass microscope slide. The slide and proscopid were positioned in the goniometer with the head centred in the yaw axis and the pitch axis centred on the eye equator. The deep pseudopupils of the eyes were viewed through a Leica MZ16 dissecting microscope. The binocular overlap was measured as the yaw angle through which the proscopid had to be rotated until the centre of one pseudopupil had reached the edge of the eye. The overlap was measured at elevations from +80 to −80°, at intervals of 5° in the range +60 to −60° and at 10° intervals outside this range (where 0° elevation corresponds to the eye equator) (see electronic supplementary material, figure 1).

### (c) Scanning electron microscopy

Dried specimens cleaned with ethanol were sputter-coated with gold and examined using a scanning electron microscope, XL-30 FEG (Philips, Eindhoven, The Netherlands).

### (d) Video analysis of gap crossing

Two horizontal wooden rods were placed a right angles within a black rectangular arena 45 × 35 cm. The positions of the horizontal rods were adjusted to create a systematic gridwork of gaps at 0, 1, 2 and 3 cm intervals in both horizontal and vertical dimensions. On some trials the horizontal target rod had an additional black band painted around it. Each proscopid (*Pr. teretrirostris*) was placed on one wooden rod and filming began, using a video camera (SONY Handycam) at 25 frames per second, once walking had started and continued until the gap was crossed. A high-speed video camera (Photron Fastcam-X 512 PCI) captured the movements during gap-crossing at 125 frames per second for off-line kinematic analysis of limb, head and body movements see electronic supplementary material, videos 1,2. The use of high intensity lighting appeared to inhibit the movements of the proscopids, although this was not studied systematically. To ensure that the proscopids would walk in the arena and perform reaches, we dimmed the lighting within the arena. Videos were saved and analysed off-line.

Once the gap-crossing was recorded, half of the proscopids had either their left or right eye occluded with black acrylic paint. The occluded eye was chosen randomly. The other half of the proscopids had the region of binocular overlap of both eyes painted with black acrylic paint. These proscopids were then replaced on the wooden beam with the same gap dimensions and their performance was again recorded. The paint was removed from the proscopids without damaging their optics or their performance (data not shown). These proscopids were then given the other occlusion, i.e. those individuals that had had a monocular occlusion had their binocular overlap regions occluded and vice versa. Again, they were replaced on the wooden beam with the same gap dimensions and their performance was recorded. There was no evidence of a change in the performance of the proscopids with repeated crossings of the gap.

### (e) Analysis

Videos were analysed in MotionScope (Redlake). All statistical comparisons between the frequencies with which a particular behavioural strategy or outcome was observed in normally sighted or occluded proscopids were by *G*-tests of independence. William's correction was used to avoid overestimation of significance when response counts are low [16]. The Dunn–Sidak correction was applied to determine significance in multiple comparisons tests [16]. All data analysis and statistics were performed using R v. 2.14.0.

## 3. Results

We walked individual proscopids (*Ps. scabra* and *Pr. teretrirostris*) along a horizontal rod placed at right angles to a second horizontal rod with an intervening gap (figure 1*c*) to determine whether they relied primarily on mechanosensory information from their forelimbs or visual information while crossing the gap. Upon arriving at the gap, the proscopids stopped walking and then, after a delay, reached directly to the rod on the opposite side. They did so without making rhythmic searching movements of the forelimbs (figure 1*d*), suggesting that their forelimbs were visually targeted. This is in contrast to stick insects or fruit flies, which invariably make rhythmic sweeping movements with their antennae or forelimbs, searching for mechanosensory cues as to the target location while crossing gaps [11,13]. Except for the smallest gaps (less than or equal to 1 × 1 cm), reaches were not made as part of ongoing walking behaviour but were distinct movements made by stationary animals.

During typical reaches the proscopids used a single forelimb, the leading limb, to reach towards and make contact with the target on the far side of the gap. The trailing forelimb then broke contact with the substrate and moved towards the target. Occasionally, both forelimbs reached for the target simultaneously. The movement of the forelimb during a reach was generated by changes in the coxal and femoro-tibial joints accompanied by movements of a highly flexible joint between the pro- and mesothorax (figure 1*d*).

**Figure 2.**
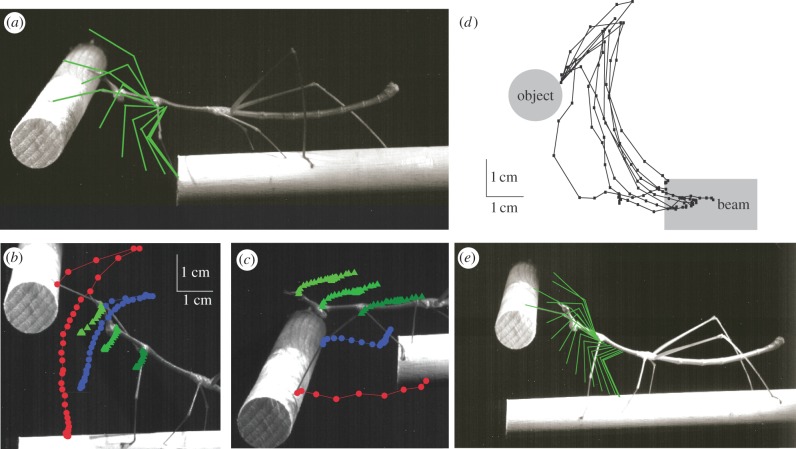
Accuracy and variability of reaching in proscopids. (*a*) Forelimb trajectory during a reach is adjusted to the location of the target. The position of the femur and tibia of the leading forelimb is indicated every 80 ms. (*b*) Postural adjustment during reaches to large vertical gaps. The positions of the tibio-tarsal (red), femero-tibial (blue), thoraco-coxal (dark green), cephalo-thoracic joints (mid green) and the eye (light green) are indicated every 80 ms. (*c*) Postural adjustment during reaches to large horizontal gaps. The positions of the tibio-tarsal, femero-tibial, thoraco-coxal, cephalo-thoracic joints and the eye are indicated every 80 ms (colour as in (*b*)). (*d*) Repeatability of reaches made to the same target location by a single *Ps. scabra*. The position of the tibio-tarsal joint of the leading forelimb is indicated every 80 ms. *n* = 8. (*e*) Reaching occurs in the absence of a horizontal gap. The position of the femur and tibia of the leading forelimb is indicated every 80 ms.

One possibility is that detecting a gap releases a generic stereotyped forelimb reaching movement that simply sweeps through the space ahead until contact is made with the target. We therefore systematically varied the separation of the horizontal rods altering the dimensions (vertical and horizontal) of the gap to test whether the forelimb trajectory is adjusted to the position of the target. This unambiguously revealed that the reaches made by proscopids are not stereotyped sweeps with a large spatial coverage but aimed at a specific target (figure 2*a–c*). During reaches across a gap in which there was a large vertical separation of the two rods (hereafter ‘vertical gaps’) the tarsus was raised above the head before being placed onto the target rod (figure 2*a,b*), whereas at gaps in which there was a large horizontal separation between the two rods (hereafter ‘horizontal gaps’) the tarsus was moved along a direct forward trajectory (figure 2*c*). For this, the entire body was lowered enabling the forelimbs to reach the target and the forelimbs were not raised above the head. When reaching across large vertical gaps, forelimb movements typically preceded movements of the head and thorax, whereas at large horizontal gaps head and thorax movements preceded those of the forelimb. The reaching motor pattern thus involves not only the forelimb joints but also the head and thorax (figure 2*b*,*c*), which are flexibly adjusted to specific visually encoded target locations. Limb movements to a particular spatial location show a high degree of repeatability, however, with the fore-tarsus showing little variation in trajectory in an experiment where proscopids crossed the same gap 8 times (figure 2*d*).

In stick insects and fruit flies, movements associated with gap crossing are initiated when the forelimb steps into a gap in the substrate [11,13]. To determine whether reaching in proscopids was triggered in a similar way or whether visual inputs were sufficient, we arranged the two horizontal rods such that they overlapped (figure 2*e*). Proscopids now reached towards the target rod before they had reached the end of the rod they walked on. Loss of forelimb contact with the substrate is therefore unnecessary for releasing the forelimb reaches of proscopids (figure 2*e*).

**Figure 3.**
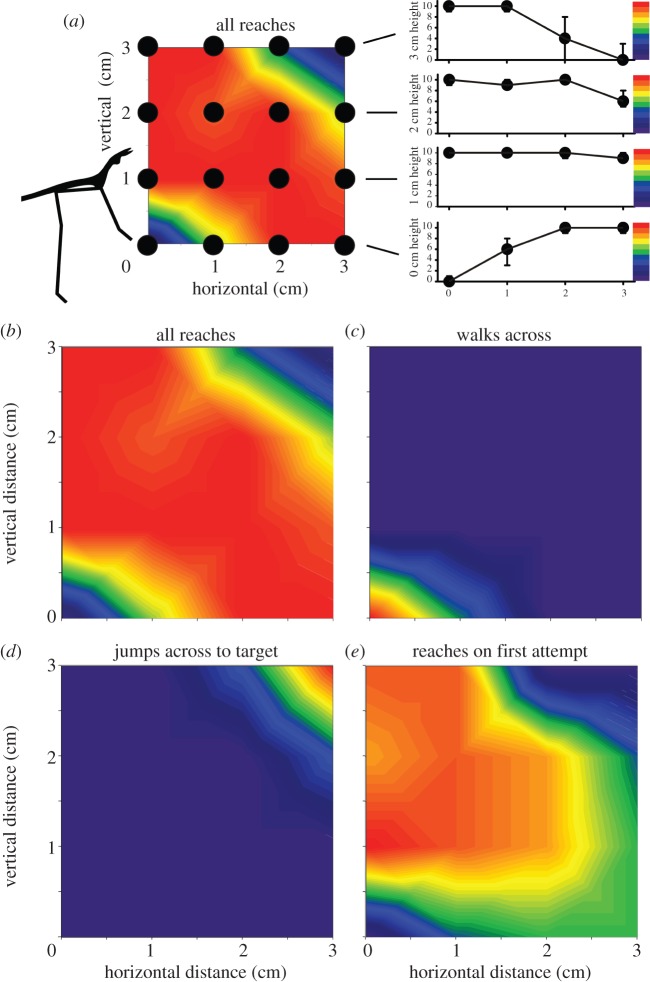
Proscopids employ different crossing behaviours depending on the width and the height of a gap. (*a*) The reaching space of *Pr. teretrirostris* with each of the target positions marked by a black circle. Each proscopid (*N* = 5) was tested at each position 10 times in a randomized order (*n* = 10). The colour maps indicate the median number of times proscopids performed a particular behaviour at a given position. The graphs to the right are transects across the reaching space at each of the height positions and the show the median and interquartile range of the number of reaches at each location. The adjacent colour charts indicate the median number of behaviours in the contour plots. The silhouette on the right is of the head and forelimbs of *Pr. teretrirostris*. (*b*) The total number of targeted reaches attempted across the reaching space. (*c*) Small gaps (less than 1 cm horizontal width and with no elevation) are mostly ignored and simply walked across. (*d*) Targeted jumps are made across the gaps with the largest horizontal and vertical distances. (*e*) Reaches where the opposite side of the gap was successfully contacted at the first attempt.

By presenting *Pr. teretrirostris* with gaps of different horizontal and vertical dimensions we were able to map the region of space in front of them in which they will reach towards a target (*N* = 5 animals, percentage out of *n* = 10 attempts at the distance by each animal) (see electronic supplementary material, table 1). This region consisted of gaps wider than 1 cm but smaller than 3 cm in both horizontal and vertical dimension (figure 3*a*). At gaps of less than 0.5 cm, 92 ± 8.2 per cent (mean ± s.e.) of proscopids simply continued to walk, ignoring the gap (figure 3*b*), although on no trial did a proscopid place its foot in the gap. With 1 cm horizontal gaps, 44 ± 4.4 per cent of proscopids walked across, the rest reached (*N* = 5, *n* = 10). At gaps greater than 2.5 cm in both vertical and horizontal distances the proportion of reaches also declined (figure 3*a*) because the proscopids made targeted jumps in 54 ± 4.3 per cent of crossings instead (figure 3*c*). In no instance was a reach attempted and then aborted in favour of a jump, demonstrating that reaches are only made to targets within range.

We assessed the accuracy of the proscopids by determining the number of reaches resulting in tarsal contact with the target. Reaches to targets within the region immediately in front of the proscopids were almost always accurate with fewer than 10 per cent (*N* = 5, *n* = 10) of reaches missing their target. However, when targets were at large horizontal distances the accuracy of reaches was diminished, so that when the target was 3 cm away 40 ± 33.1 per cent of proscopids took two or more attempts to successfully contact it, the tarsus returning to its resting position between attempts (figure 3*d*; *N* = 5, *n* = 10). Nevertheless, even at these gaps, the proscopids were always able to reach successfully and in no case was reaching aborted after an initial miss.

**Figure 4.**
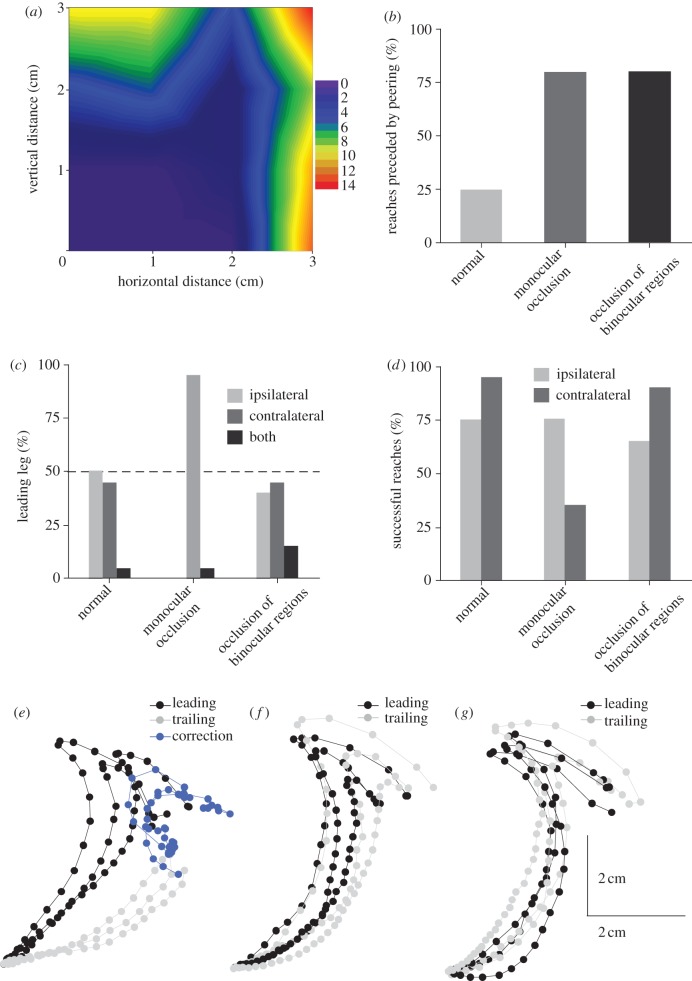
Monocular visual inputs obtained before the initiation of reaching are sufficient for accurate targeting of the leading forelimb. (*a*) Peering normally occurs at horizontal and vertical gaps greater than 2 cm. *N* = 6 *Pr. teretrirostris*, *n* = 10 reaches per animal. (*b*) Occlusion of visual inputs increases the frequency of peering preceding reaches at small gaps. *N* = 20 normally sighted *Ps. scabra*, *N* = 20 monocularly occluded and *N* = 20 binocularly occluded reaches. (*c*) Monocular occlusion but not occlusion of the binocular overlap regions of both eyes reduces the frequency with which the ipsilateral forelimb is used at the leading leg. *N* = 20 normally sighted *Ps. scabra*, *N* = 20 monocularly occluded and *N* = 20 binocularly occluded reaches. (*d*) Monocular occlusion but not occlusion of the binocular overlap regions of both eyes reduces the accuracy with which the ipsilateral forelimb is targeted. *N* = 20 normally sighted *Ps. scabra*, *N* = 20 monocularly occluded and *N* = 20 binocularly occluded reaches. (*e*) Trajectories of the leading (black) and trailing (grey) legs in monocularly occluded *Ps. scabra*. The initial trajectory is shown in light grey, the compensatory movement of the leg around the target is shown in blue. (*f*) Trajectories of the leading and trailing legs in normally sighted *Ps. scabra*. (*g*) Trajectories of the leading and trailing legs following bilateral occlusion of the binocular overlap regions in *Ps. scabra*.

How do proscopids judge the target's distance? One way in which insects, including locusts and mantids, assess object distance is by making side-to-side peering movements that generate motion parallax [17–19]. Proscopids likewise peer when facing an object and could therefore use motion parallax to determine target distance. Peering in proscopids was, however, restricted to more distant targets (figure 4*a*); at smaller gaps (*Pr. teretrirostris*; less than 2 × 2 cm; *Ps. scabra*; less than 2.5 × 2.5 cm), less than 25 per cent of reaches were preceded by peering. At the most extreme vertical distance approximately 64 ± 34.3 per cent of reaches were preceded by peering; at the most extreme horizontal distances all reaches were preceded by at least one bout of peering (figure 4*a*).

For distance estimation through motion parallax, monocular cues suffice. Other monocular cues can be used to estimate object distance indirectly from retinal elevation [20–22] or from looming cues [23]. Proscopids were able to make direct, accurate reaches across gaps of differing horizontal and vertical distances to horizontal rods at different elevations (figure 3*d*). Thus, the proscopids cannot be using the retinal elevation to judge the distance of the object. Although looming cues could be used to estimate object distance during reaching, proscopids are typically stationary prior to reaching and their head movements during reaching do not produce a pure looming cue (figure 2*b*,*c*). The compound eyes of the proscopids were also at different distances from the object when reaches were made (data not shown). Thus, proscopids do not rely on retinal elevation or looming cues to estimate object distance.

Insects can also use binocular visual cues to estimate distance [24]. We fully occluded one of the compound eyes (in *Ps. scabra*) to determine whether the proscopids used monocular or binocular cues. Following monocular occlusion, the number of reaches at gaps (less than or equal to 2.5 × 2.5 cm) that were preceded by peering increased to 80 per cent, a significantly higher proportion than less than 25 per cent in fully sighted proscopids (figure 4*b*) (reaches preceded by peering; *G*-test; *G* = 12.91; 1 d.f.; *p* < 0.001; *N* = 40, 20 fully sighted and 20 occluded animals, 1 trial per animal, in all subsequent statistical tests). This suggests that monocular occlusion interfered with the mechanism normally used to estimate target distance across small gaps. Thus, in the absence of binocular cues, proscopids resort to peering to generate the motion parallax necessary for distance estimation even at small gaps.

Following monocular occlusion, reaches were made almost exclusively with the forelimb of the sighted side as the leading limb (ipsilateral versus contralateral forelimb leading; *G*-test; *G* = 14.42; 1 d.f.; *p* < 0.001; *N* = 40), whereas in fully sighted animals both forelimbs were equally likely to lead (figure 4*c*). The accuracy of the leading forelimb on the sighted side was unimpaired by the monocular occlusion (figure 4*d*) (frequency of directly targeted *versus* corrected reaches; *G*-test; *G* = 0; 1 d.f.; n.s.; *N* = 40). However, the trailing forelimb on the occluded side was significantly impaired in accuracy (figure 4*d*); it was raised to the underside of the target and then moved to the front in a series of corrective movements (figure 4*e*) (frequency of directly targeted versus corrected reaches; *G*-test; *G* = 18.06; 1 d.f.; *p* < 0.001; *N* = 40). This contrasts with fully sighted individuals, in which the trailing leg was moved in a direct trajectory similar to the leading leg (figure 4*f*). Thus, motion parallax from one compound eye is sufficient to allow the accurate placement of the ipsilateral but not of the contralateral leg. Moreover, mechanosensory information from the leg that has already made contact with the target is not sufficient to permit the trailing forelimb to be accurately targeted.

The increased use of peering to generate motion parallax after monocular occlusion suggested that normally another distance estimation mechanism is used, one that relies on binocular cues. We therefore occluded the frontal region of binocular overlap on both compound eyes to eliminate binocular cues, but permit monocular cues from both eyes. Following this frontal binocular occlusion, 80 per cent of reaches were again preceded by peering—a significantly higher proportion than in normally sighted individuals (figure 4*b*) (reaches preceded by peering; *G*-test; *G* = 12.91; 1 d.f.; *p* < 0.001; *N* = 40). This suggests that both full monocular and frontal binocular occlusions disrupt a binocular cue involved in distance estimation, but that proscopids can in both cases use peering as an alternative strategy to generate depth cues.

Removing the region of binocular overlap neither reduced the accuracy of the reach significantly (frequency of directly targeted versus corrected reaches; *G*-test; *G* = 0.48; n.s.; *N* = 40) nor affected which leg was used to reach (figure 4*c*,*d*) (ipsilateral versus contralateral forelimb leading; *G*-test; *G* = 0.09; 1 d.f.; n.s.; *N* = 40). Moreover, unlike after monocular occlusion, the accuracy of the trailing leg was not significantly affected following binocular occlusion (figure 4*g*) (frequency of directly targeted versus corrected reaches; *G*-test; *G* = 0.37; 1 d.f.; n.s.; *N* = 40). This demonstrates that binocular cues are not necessary for an accurate placement. Thus, fully sighted proscopids appear to rely on binocular cues but following disruption of binocular vision they can use monocular cues including motion parallax through peering. In those instances where no peering was observed, it is possible that the animals made use of parallax cues generated through incidental movements of the head while walking [9,11].

## 4. Discussion

We have shown that proscopid grasshoppers are capable of making visually targeted reaches to discrete locations in space. Four lines of evidence support this conclusion: (i) no contact is made with the target by the antennae prior to the execution of a reach, (ii) each reach is a single, smooth movement of the forelimb towards the target and does not involve the sampling of space through repeated searching movements, (iii) the trajectory of the reach is adjusted to the position of the target, (iv) monocular occlusion prevents accurate reaching by the ipsilateral forelimb and suppresses its use. Stationary proscopids made reaches to nearby targets with their forelimbs but reaches to more distant targets incorporated middle and hind limb movements that adjusted the thorax and abdomen positions, a process that in humans has been termed ‘reaching beyond reach’ [25].

The visually targeted reaches of proscopids are remarkable in two regards: the high accuracy and the flexible switching between alternative distance estimation mechanisms. Our data suggest that proscopids use binocular cues for distance estimation when reaching across small gaps, but at larger gaps they use peering to estimate target distance from motion parallax. Both of these strategies are used by other insects; praying mantises (e.g. *Mantis religiosa*), and possibly ground beetles (*Asaphidion flavipes*), also estimate the distance of nearby targets using binocular cues [24,26,27], while locusts and mantises use side-to-side translational head movements to obtain motion parallax [17–19,28,29]. Unlike these previously studied insect species, however, proscopids switch flexibly between the two mechanisms in the same behavioural context, depending not only on target distance, but also when challenged by partial occlusion of the visual field. After occlusion of binocular overlap regions, accuracy of the forelimb placement remained unimpaired as the proscopids compensated by using monocular cues, including motion parallax. By contrast, mantises do not use monocular cues to compensate for the loss of binocular cues, possibly because the two strategies are tied to two distinct behavioural contexts: they use binocular cues to estimate the distance of moving objects and motion parallax for stationary objects [30]. Locusts, likewise, do not compensate for the occlusion of their binocular overlap regions with motion parallax [9], possibly reflecting differences in the absolute distances and accuracy of the estimates needed for forelimb targeting in locusts and proscopids.

Following monocular occlusion, proscopids again adjusted their behaviour by switching to peering, and by preferentially reaching with the forelimb ipsilateral to the unoccluded eye, which they placed with undiminished accuracy. They were then, however, unable to accurately place the limb ipsilateral to the occlusion, as are locusts following monocular occlusion [9]. Thus, monocular visual information from the unoccluded eye and mechanosensory feedback from the already targeted forelimb are insufficient to accurately target the contralateral forelimb. This suggests either that monocular distance estimates cannot be transferred to the contralateral forelimb controller, or that corrective visual feedback from the ipsilateral compound eye is necessary for accurate placement.

In grasshoppers then, such as proscopids and locusts, monocular cues seem to descend only to the ipsilateral prothoracic limb controller, with little proprioceptive information flow between forelimbs on the same segment, despite the numerous proprioceptors monitoring their position and movement (for a review see [31]). Indeed, while ablation of inputs from one proprioceptor, the femoral chordotonal organ, reduces the accuracy of that forelimb, it does not affect the contralateral forelimb [9]. Whether this separation of the information transmitted to and between the forelimb controllers occurs in other insects is unknown. In proscopids, however, a flexible combination of visual cues can be used to judge distance, but this is coupled to a more or less rigid partition between left- and right-handed proprioceptive and motor control circuitry in the thoracic ganglia.

Fruit flies (*Drosophila melanogaster*) also use visual inputs during gap crossing, which is initiated only if they can see an opposing side [11]. However, to find a foothold on the opposite side, these flies use rhythmic ‘leg over head’ movements that are not targeted directly to a specific location. Both mantises and mantispids have evolved modified forelimbs for the execution of raptorial strikes to small moving targets, a remarkable example of morphological and behavioural convergence [32,33]. Unlike the proscopids' reaches, however, their raptorial strikes involve a series of stereotyped forelimb movements, in which the femoro-tibial joints are extended and then rapidly closed, trapping anything within a wide arc of space. Nevertheless, the strike's direction can be adjusted through postural changes [33], which resemble those made by the proscopids when reaching. Whether the raptorial forelimb strikes/sweeps can be targeted in the same way as the proscopids' reaches is unclear, but the need to trap prey between the femur and tibia, which are both long segments, contrasts with the accurate tarsal placement of *Proscopia* and may not require such accurate control.

Our study also demonstrates how parallel evolution of a similar overall life style can result in radically different solutions in different taxa. Proscopids and stick insects have converged upon similar strategies for remaining concealed within the arboreal environment [12]; both have narrow, elongated tubular bodies and limbs, and colouration resembling the plants on which they climb. Although they must solve similar tasks, stick insects cross gaps by using their antennae and forelimbs to sweep through and sample the space ahead of them, the resulting mechanosensory feedback allowing them to gauge the distance and location of the target [13], whereas proscopids use visual cues. This difference is reflected in their relative investment in their visual and mechanosensory systems; stick insects have relatively small eyes and large antennae while the reverse is true for proscopids. Indeed, about two-thirds of the volume of the proscopids brain is occupied by the optic lobes (J.E.N., S.R.O. & S.M.R. 2007, unpublished data). One explanation for proscopids' use of visual cues is that their ancestors already possessed relatively large visual systems and small antennae. Extant grasshoppers (Caelifera) typically have short antennae and large eyes in contrast to most other Orthopteroid insects such as stick insects (Phasmatodea), cockroaches (Blattodea) and mantophasmids, but also crickets and bush crickets (Ensifera) within the Orthoptera itself. Indeed, visually targeted reaching in proscopids may be evolved from the visual adjustment of forelimb movements while walking, which has been shown in locusts walking along horizontal ladders [9]. A similar scenario has been evoked to explain the evolution of visually guided reaching in primates from visual targeted forelimb movements during walking in other mammals [6].

The accuracy and flexibility of the visually targeted reaches of the proscopid is remarkable considering the intrinsic difficulty of the task. Visual targeting of forelimbs requires that the position of an object is located in space and a motor pattern planned that moves the limb to the object. The object's position is encoded within a retinotopic co-ordinate frame that must be transformed to generate the motor activity necessary to target the multi-jointed forelimb and, for reaches to more distant objects, the movements of all six legs. There is considerable debate regarding the transformations involved in human and primate reaching and numerous brain regions have been implicated (for a review see [8]). Our study cannot determine what kind of co-ordinate transformation proscopids make during reaching, although in many cases insects (and other animals) can perform behaviours that appear to require numerous computations by using heuristics that reduce the computational burden. Such assumptions are thought to underlie many behaviours including the classification of conspecifics versus predators in fiddler crabs [22] or prey in jumping spiders [34,35], the interception of female hoverflies by males [36], the size of potential nest sites in ants [37] and numerous problems related to spatial orientation [38]. Electrophysiological analyses of the circuits underlying reaching in proscopids may thus throw light on computationally light solutions to visually targeted movements.
